# Mediating Effect of Internet Addiction on the Relationship Between Individualism and Cyberbullying: Cross-Sectional Questionnaire Study

**DOI:** 10.2196/16210

**Published:** 2020-05-28

**Authors:** Ibrahim Arpaci, Thabet Abdeljawad, Mustafa Baloğlu, Şahin Kesici, Ibrahim Mahariq

**Affiliations:** 1 Department of Computer Education and Instructional Technology Tokat Gaziosmanpasa University Tokat Turkey; 2 Department of Mathematics and General Sciences Prince Sultan University Riyadh Saudi Arabia; 3 Department of Medical Research China Medical University Taichung Taiwan; 4 Department of Computer Science and Information Engineering Asia University Taichung Taiwan; 5 Department of Special Education Faculty of Education Hacettepe University Ankara Turkey; 6 Department of Educational Sciences Necmettin Erbakan University Konya Turkey; 7 College of Engineering and Technology American University of the Middle East Kuwait

**Keywords:** vertical individualism, horizontal individualism, cyberbullying, internet addiction

## Abstract

**Background:**

Among a variety of dynamics that may have effects on internet-related behaviors, cultural orientation is particularly important. Previous studies suggest that individualism is a strong determinant of certain behaviors. In addition, findings suggest that vertical individualism may lead to the development of more tolerance for addiction and aggression on the internet.

**Objective:**

This study aimed to investigate whether vertical individualism has significant positive effects on cyberbullying and internet addiction and whether horizontal individualism has significant negative effects on cyberbullying and internet addiction. A theoretical model was specified to test the relationships among vertical versus horizontal individualism, cyberbullying, and internet addiction.

**Methods:**

A total of 665 college students were selected using a convenience sampling method and willingly participated in the study. Participants’ ages ranged from 17 to 19 years (mean 17.94 years, SD 1.12 years). Of the group, 462 were women (462/665, 69.5%), and 203 were men (203/665, 30.5%). Study majors represented were mathematics (113/665, 17%), sciences (102/665, 15.3%), instructional technology (99/665, 14.9%), psychology (98/665, 14.7%), and others (253/665, 38.1%). Self-report instruments were used to measure vertical/horizontal individualism, cyberbullying, and internet addiction.

**Results:**

Results show a significant positive effect of vertical individualism (effect size 0.10) and significant negative effect of horizontal individualism (effect size –0.12) on cyberbullying. In addition, the direct effect of vertical individualism on internet addiction was significant (effect size 0.28), but the direct effect of horizontal individualism was not (effect size –0.05). Internet addiction had a significant direct effect on cyberbullying (effect size 0.39) as well as an intervening effect on the relationship between vertical individualism and cyberbullying. Results also indicate significant gender differences in cultural patterns and internet addiction.

**Conclusions:**

The findings suggest that horizontal and vertical individualism have significant effects on internet addiction. The findings also suggest that vertical individualists are more vulnerable to internet addiction. Further, the findings indicate a significant relationship between internet addiction and cyberbullying.

## Introduction

### Background

Among a variety of dynamics that may have effects on internet-related behaviors, cultural orientation is particularly noteworthy [1-4]. Hofstede [5] identified four key cultural orientations of people, among which individualism versus collectivism is one of the rather more frequently investigated dimensions [5-8]. In his conceptualization, individualism is defined as the tendency to which “an individual is supposed to take care of himself/herself” [9], and collectivism is the tendency “to which an individual remains integrated into a group” [10]. Triandis [11] argued that individualism and collectivism emerge from status-equal (ie, horizontal) versus status-unequal (ie, vertical) relationships and therefore identified horizontal versus vertical individualism and horizontal versus vertical collectivism. Our study focused on the effects of horizontal versus vertical individualism.

Triandis [11] argued that individualists tend to emphasize an autonomous self-concept, whereas collectivists are inclined to consider themselves as a part of the group. Therefore, an independent versus interdependent self is one of the distinctive characteristics of the two [10]. Contrary to collectivists, individualists prioritize personal goals over the group goals. Internal processes such as attitudes predict social behaviors among individualists. However, among collectivists, social behaviors are predicted by subjective norms, obligations, and perceived duties [12]. Individualists tend to drop a relationship when the cost of the relationship exceeds their personal benefits; however, collectivists try maintaining the relationship even if the cost surpasses their personal benefits [13].

Triandis [11] also identified additional personality characteristics that differentiate between horizontal individualism and vertical individualism. He suggested that vertical individualism defines the self as autonomous, different, and unequal in status with others. Competition is one of the key aspects of vertical individualism. On the other hand, horizontal individualism defines the self as autonomous and independent but also equal to the self of others. Floros et al [14] found that internet addicts exhibit higher impulsivity and help-rejecting behaviors, suggesting that character and personality are significant factors in predicting internet addiction.

Brady [15] described cyberbullying as “the use of communication-based technologies including social networking sites to engage in deliberate harassment or intimidation of other individuals or groups of persons using online speech or expression.” Contemporary research shows that cyberbullying is an increasingly epidemic problem among children [16] as well as adolescents [17]. Casas et al [18] found that bullying is strongly influenced by personal and contextual factors. For example, they argue that empathy was a significant predictor of cyberbullying. Similarly, Mishna et al [19] suggested two main risk factors for involvement in cyberbullying: the increasing use of technology [20] and the lack of face-to-face interactions associated with social cues [21,22]. In addition, Smith et al [23] found that being a cyber-victim is correlated with internet use. That is, the more intensive use of the internet, the higher the likelihood of cyberbullying. Therefore, we decided to investigate the effect of internet addiction on cyberbullying.

Based on the cognitive-behavioral model of Davis [24], internet addiction is conceptualized as “an impulse control disorder” [25] and found to be related to a wide range of psychosocial complications [26,27], including cyberbullying [28,29]. Internet addiction or problematic internet use [30] is one of the central research areas for college students. In addition, assessment instruments [31] and screening methods [32] have been developed in the area of problematic internet use or internet addiction. However, the potential association between internet addiction and individuals’ cultural orientations (ie, individualism) has not been sufficiently investigated. In general, previous studies showed positive associations between individualism and addictive behavior [33,34] or aggressive behavior [35]. Accordingly, we focused on the effect of the relationship between vertical versus horizontal individualism and internet addiction on cyberbullying.

Cyberbullying and internet addiction have been relatively more frequent themes of recent research [36,37]. However, the lack of previous studies on the impact of cultural individualism on cyberbullying or internet addiction calls for an investigation [38]. Therefore, the purpose of this research was to fill the gap in prior studies by studying the role of internet addiction in the association between vertical versus horizontal individualism and cyberbullying. Such an investigation is warranted for the screening, identification, diagnosis, prevention, and treatment of cyberbullying and internet addiction.

### Hypotheses

In general, individualists tend to behave autonomously and prioritize their personal preferences [39]. Hooker [40] argues that individualists have a stronger sense of private space and are more likely to prefer loose personal ties [41]. However, previous studies have reported significant individual differences between horizontal individualists and vertical individualists [42-44].

Horizontal individualism is described as “a model of independent self that fosters a propensity to value uniqueness and social equality,” whereas vertical individualism describes “an autonomous self that garners gratification through competition and personal achievement” [8,11]. Vertical individualists are particularly concerned with comparing themselves with others and are likely to enjoy “competition, hedonism, and acquiring status through rivalry” [45]. Vertical individualists prefer to accept inequality and acknowledge the importance of status as well as social rank, whereas horizontal individualists prefer to accept interdependence and equal status for all [11]. We deduced from previous studies that vertical individualists emphasize competition, prestige, hedonism, and status more than horizontal individualists [8].

Previous studies on substance dependence suggested a positive correlation between individualism and addictive behavior [33]. In addition, Bergmüller [35] found that individualism is a strong determinant of aggressive behavior. Ogihara and Uchida [46] found that individualism is negatively related to the number of intimate friends and subjective well-being. These findings suggest that vertical individualism may lead to the development of more tolerance for addiction and aggression. Thus, we theorized that people who score higher for vertical individualism would be more inclined to cyberbullying and internet addiction. We hypothesized that vertical individualism would have a significant positive effect on cyberbullying (H1a) and internet addiction (H1b), whereas horizontal individualism would have a significant negative effect on cyberbullying (H2a) and internet addiction (H2b).

Social repercussions are among the most negative consequences of internet addition [47]. Ko et al [48] suggested that individuals with internet addiction are more likely to have aggressive behaviors. Recent studies also showed significant relationships between internet addition and cyberbullying [28,29]. For example, Gámez-Guadix et al [49] found that cyberbullying was predicted by problematic internet use. You and Lim [29] and Chang et al [50] suggested that internet addiction is associated with cyberbullying. Therefore, we hypothesized that the degree of internet addiction would be positively related to cyberbullying [H3].

**Figure 1:**
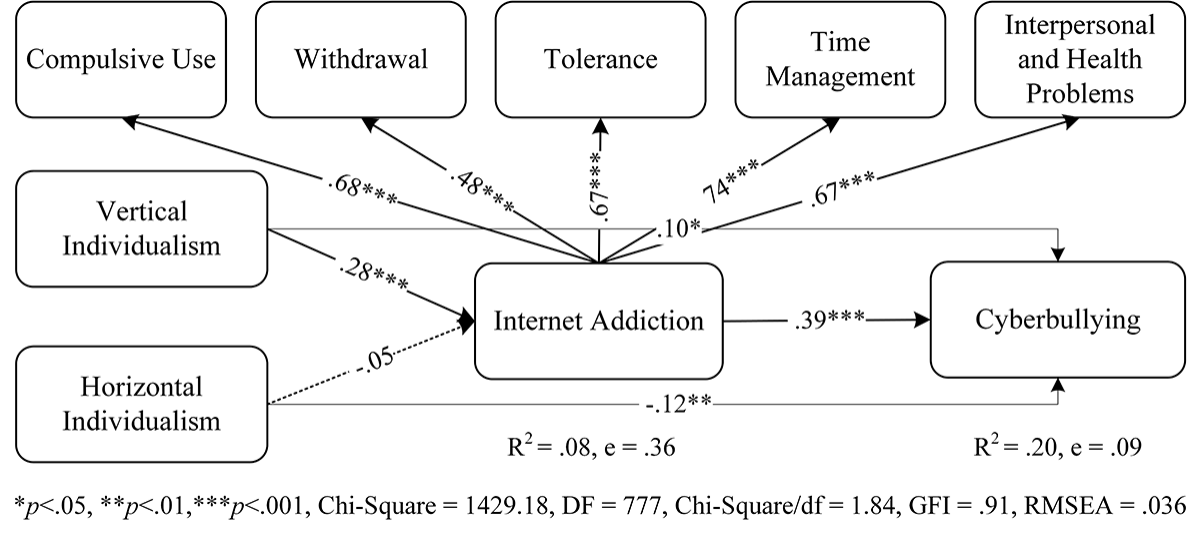
Relationships between vertical-horizontal individualism, cyberbullying, and internet addiction.

According to Hofstede [5], individuals may behave differently depending on their cultural orientations. Therefore, we used horizontal and vertical individualism as extraneous variables in the theoretical model shown in Figure 1. Further, we included internet addiction as an intervening variable between individualism and cyberbullying.

## Methods

### Participants

A total of 665 freshmen from two state universities in the central part of Turkey who were selected using a convenience sampling method willingly participated in the study. The participants completed an anonymous online survey and received extra course credit for participation. Participants’ ages ranged from 17 to 19 years (mean 17.94 years, SD 1.12 years). Of the group, 462 were women (462/665, 69.5%), and 203 were men (203/665, 30.5%). Students from mathematics (113/665, 17%), science (102/665, 15.3%), instructional technology (99/665, 14.9%), psychology (98/665, 14.7%), and other departments (253/665, 38.1%) were represented in the study.

### Measures

We used a total of 67 items: 18 items for individualism (10 items for horizontal individualism and 8 items for vertical individualism), 23 items for cyberbullying, and 26 items for internet addiction. All instruments asked participants to rate their level of agreement using a 5-point Likert scale ranging from “strongly disagree” to “strongly agree.”

### The Individualism-Collectivism Scale

Singelis et al [8] developed the Individualism-Collectivism scale to examine differences in vertical versus horizontal individualism and vertical versus horizontal collectivism. Evidence for the validity and reliability of the scale has been documented [8]. Of the 18 individualism items, 8 items measure vertical individualism, and 10 items measure horizontal individualism (for sample items, see Table 1). Wasti and Erdil [51] adapted the scale into Turkish and reported that the Cronbach α internal consistency coefficients were .67 for horizontal individualism and .73 for vertical individualism. However, in this study, we obtained Cronbach α values of .81 and .82 for horizontal and vertical individualism, respectively.

**Table 1:** Evidence of the validity and reliability of the measures used in the study.

Construct, Sample item		α^a^	Item-total correlation^b^	Factor loading^b^	Communality^b^	Total variance explained^a^
**Cyberbullying**						
	CB1	N/A	.67	.69	.48	N/A
	CB2: I create accounts in websites, such as Facebook and Twitter, secretly using others’ names	N/A	.83	.84	.71	N/A
	CB3	N/A	.84	.86	.74	N/A
	CB4	N/A	.89	.90	.82	N/A
	CB5	N/A	.86	.88	.78	N/A
	CB6	N/A	.87	.89	.79	N/A
	CB7	N/A	.78	.81	.65	N/A
	CB8	N/A	.90	.92	.84	N/A
	CB9	N/A	.87	.89	.78	N/A
	CB10	N/A	.87	.89	.79	N/A
	CB11	N/A	.83	.84	.71	N/A
	CB12	N/A	.84	.86	.74	N/A
	CB13	N/A	.88	.90	.80	N/A
	CB14	N/A	.86	.87	.76	N/A
	CB15	N/A	.74	.75	.57	N/A
	CB16	N/A	.76	.77	.60	N/A
	CB17	N/A	.89	.91	.82	N/A
	CB18	N/A	.74	.76	.57	N/A
	CB19	N/A	.84	.86	.74	N/A
	CB20	N/A	.85	.87	.75	N/A
	CB21	N/A	.88	.90	.81	N/A
	CB22	N/A	.82	.84	.70	N/A
	CB23	N/A	.72	.74	.55	N/A
	Total subscale	.98	N/A	N/A	N/A	69.57
**Compulsive use**						
	CU1: I can’t control myself when it comes to the internet	N/A	.78	.78	.61	N/A
	CU2	N/A	.79	.75	.54	N/A
	CU3	N/A	.77	.82	.67	N/A
	CU4	N/A	.80	.40	.49	N/A
	CU5	N/A	.78	.79	.62	N/A
	Total subscale	.82	N/A	N/A	N/A	58.84
**Withdrawal**						
	W1: If I don’t use the internet, I feel uncomfortable	N/A	.83	.82	.68	N/A
	W2	N/A	.84	.80	.63	N/A
	W3	N/A	.86	.73	.54	N/A
	W4	N/A	.83	.81	.66	N/A
	W5	N/A	.82	.86	.73	N/A
	Total subscale	.86	N/A	N/A	N/A	64.68
**Tolerance**						
	T1: I spend more time on the internet than I expect	N/A	.85	.83	.69	N/A
	T2	N/A	.83	.87	.76	N/A
	T3	N/A	.82	.89	.78	N/A
	T4	N/A	.86	.82	.68	N/A
	Total subscale	.88	N/A	N/A	N/A	72.82
**Time management**						
	TM1: I use the internet during my sleeping time		.85	.82	.67	N/A
	TM2	N/A	.84	.83	.70	N/A
	TM3	N/A	.84	.85	.71	N/A
	TM4	N/A	.87	.75	.57	N/A
	TM5	N/A	.84	.84	.71	N/A
	Total subscale	.88	N/A	N/A	N/A	67.20
**Interpersonal and health problems**						
	P1: I neglect my family because of the internet	N/A	.90	.80	.65	N/A
	P2	N/A	.90	.80	.64	N/A
	P3	N/A	.90	.74	.55	N/A
	P4	N/A	.89	.85	.72	N/A
	P5	N/A	.89	.84	.70	N/A
	P6	N/A	.89	.85	.73	N/A
	P7	N/A	.90	.77	.59	N/A
	Total subscale	.91	N/A	N/A	N/A	65.31
**Horizontal individualism**						
	HI1	N/A	.79	.75	.57	N/A
	HI2	N/A	.79	.52	.40	N/A
	HI3: I often do my own thing	N/A	.80	.62	.44	N/A
	HI4	N/A	.78	.61	.52	N/A
	HI5: I like my privacy	N/A	.78	.79	.68	N/A
	HI6	N/A	.78	.65	.51	N/A
	HI7	N/A	.78	.67	.51	N/A
	HI8	N/A	.80	.77	.59	N/A
	HI9	N/A	.80	.64	.41	N/A
	HI10	N/A	.80	.67	.46	N/A
	Total subscale	.81	N/A	N/A	N/A	50.89
**Vertical individualism**						
	VI1: Winning is everything	N/A	.80	.84	.79	N/A
	VI2	N/A	.81	.90	.83	N/A
	VI3	N/A	.81	.46	.42	N/A
	VI4	N/A	.80	.81	.70	N/A
	VI5	N/A	.81	.87	.81	N/A
	VI6: When another person does better than I do, I get tense and aroused	N/A	.80	.83	.76	N/A
	VI7	N/A	.80	.81	.72	N/A
	VI8	N/A	.81	.86	.81	N/A
	Total subscale	.82	N/A	N/A	N/A	72.98

^a^Calculated for the subscale only.

^b^Calculated for the subscale items only.

### The Internet Addiction Scale

Internet addiction levels were measured using the Internet Addiction Scale [52]. This 26-item scale includes 5 subscales: “compulsive use,” “withdrawal,” “tolerance,” “time management problems,” and “interpersonal and health problems” (for sample items, see Table 1). The scale was adapted into Turkish by Kesici and Sahin [53], where they reported satisfactory reliability and validity properties of the Turkish scale. The Cronbach α coefficient of the total scale was .88, and factor loadings ranged from .44 to .74. Likewise, we calculated a Cronbach α value of .86 for the total scale.

### The Cyberbullying Scale

Cyberbullying was measured using 23 items [54]. The authors reported that the Cronbach α coefficient of the single-factor scale was .95. We also found a Cronbach α value of .98 for the scale.

## Results

### Descriptive Findings

Almost all participants had a smartphone, and approximately two-thirds had a notebook. Further, 543 students (543/665, 81.7%) used technology more than 4 hours a day, and 433 students (433/665, 65.1%) used the internet more than 4 hours a day. Pearson correlation analyses showed a significant correlation between internet use and both vertical individualism (r=.11, *P*=.01) and horizontal individualism (r=.09, *P*=.02). In addition, all subscales of the Internet Addiction Scale were significantly positively correlated with cyberbullying. Table 2 reports the descriptive statistics computed on the study variables.

**Table 2:** Descriptive statistics, bivariate correlations, and principal component analysis.

Variable		1. Cyberbullying	2A. Internet addiction: compulsive use	2B. Internet addiction: withdrawal	2C. Internet addiction: tolerance	2D. Internet addition: time management	2E. Internet addiction: interpersonal health problems	3. Horizontal individualism	4. Vertical individualism
**1. Cyberbullying**									
	r		.29	.31	.28	.44	.42	–.01	.17
	*P* value		.000	.000	.000	.000	.000	.710	.000
**2A. Internet addiction: compulsive use**									
	r	.29	—^a^	.72	.74	.66	.61	.14	.23
	*P* value	.000	—	.000	.000	.000	.000	.000	.000
**2B. Internet addiction: withdrawal**									
	r	.31	.72	—	.67	.61	.56	.13	.23
	*P* value	.000	.000	—	.000	.000	.000	.001	.000
**2C. Internet addiction: tolerance**									
	r	.28	.74	.67	—	.71	.65	.12	.25
	*P* value	.000	.000	.000	—	.000	.000	.003	.000
**2D. Internet addition: time management**									
	r	.44	.66	.61	.71	—	.84	.06	.23
	*P* value	.000	.000	.000	.000	—	.000	.149	.000
**2E. Internet addiction: interpersonal health problems**									
	r	.42	.61	.56	.65	.84	—	.05	.24
	*P* value	.000	.000	.000	.000	.000	—	.185	.000
**3. Horizontal individualism**									
	r	–.01	.14	.13	.12	.06	.05	—	.40
	*P* value	.710	.000	.001	.003	.149	.185	—	.000
**4. Vertical individualism**									
	r	.17	.23	.23	.25	.23	.24	.40	—
	*P* value	.000	.000	.000	.000	.000	.000	.000	—
Mean		26.71	10.37	9.68	8.22	7.77	10.81	40.01	25.47
SD		8.58	3.24	3.54	2.98	3.32	4.37	5.40	5.84
Minimum- Maximum		24-96	5-20	5-20	4-16	5-20	7-28	10-50	8-40
Skewness (SE .10)		.12	.62	.84	.55	.50	.48	–.71	–.09
Kurtosis (SE .19)		1.03	.19	.25	–.22	.96	.03	.97	.20
KMO^b^		.97	.83	.86	.81	.85	.91	.87	.80
**Chi-square**									
	χ2	19,202	1108	1453	1371	1667	2872	1749	1907
	df	276	19	10	6	10	21	45	28
	*P* value	.000	.000	.000	.000	.000	.000	.000	.000

^a^Not applicable.

^b^Kaiser-Meyer-Olkin

### Validity and Reliability

Prior to the analyses, data were checked for the adequacy of factor analysis [55,56]. Table 2 also shows the suitability of the data for factor analysis. An exploratory factor analysis was employed by using principal component extraction to assess the construct validity of the scales. The percentages of total variance explained ranged from 50.89% to 72.98%, which are higher than the acceptable minimum value of .40 [57,58]. Each measurement item has a factor loading above .40 and a communality value above .40 [59,60]. The corrected item-total correlation coefficients ranged from .67 to .90, indicating moderate to high homogeneity. Cronbach α coefficients ranged from .81 to .98, indicating good to very good internal consistency [61]. Validity and reliability results are presented in Table 1.

### Common Method Bias

Harman’s one-factor test was used to check common method bias [62]. All dependent and independent variables were subjected to the exploratory factor analysis. The factors together accounted for 64.30% of the total variance, while the first factor explained only 24.96%. These findings suggested that common method bias was not a concern in the data set.

### Structural Model

It was theorized that internet addiction would serve as an intervening variable between vertical versus horizontal individualism and cyberbullying. Structural equation modeling was conducted via maximum likelihood to test the model. Results show that the structural model produced acceptable fit indices (Table 3).

The direct effects of vertical individualism on cyberbullying (β=.10, critical ratio [CR]=2.31, *P*=.02) and internet addiction (β=.28, CR=5.81, *R^2^*=.08, *P*<.001) were positively significant. The direct effect of horizontal individualism on cyberbullying (β=–.12, CR=–2.96, *P*=.01) was significant, but it was not significant on internet addiction (β=–.05, CR=–1.08, *P=*.32). Therefore, the null hypotheses for H1a, H1b, and H2a were rejected, but H2b failed to be rejected. The proposed path coefficient between internet addiction and cyberbullying was also positive and significant (β=.39, CR=3.72, *P*<.001). The effect size in this relationship was *R*^2^=.18. Therefore, the null hypothesis for H3 was also rejected. Figure 1 shows the results of the structural equation modeling analysis, including standardized path coefficients and significance levels along with the *R*-squared values and respective error terms.

**Table 3:** Model fit indices of the structural model.

Indices	Model	Acceptable values
χ^2^	1429.18	N/A^a^
*P* value	<.001	.05≤*P*≤1.00 [63]
χ^2^_df_	1.84	<3 [64]
GFI^b^	.91	≥.90 [65]
AGFI^c^	.89	≥.80 [66]
SRMR^d^	.06	≤.10 [64]
RMR^e^	.05	<.05 [67]
RMSEA^f^	.04	<.08 [65]
NFI^g^	.93	≥.90 [65]
TLI^h^	.96	≥.90 [68]
CFI^i^	.96	≥.90 [69]
IFI^j^	.96	≥.90 [70]

^a^A recommended threshold or acceptable value does not exist.

^b^GFI: goodness of fit index.

^c^AGFI: adjusted goodness of fit index.

^d^SRMR: standardized root mean square residual.

^e^RMR: root mean square residual.

^f^RMSEA: root mean square error of approximation.

^g^NFI: normed fit index.

^h^TLI: Tucker Lewis index.

^i^CFI: comparative fit index.

^j^IFI: incremental fit index.

### Mediation Analysis

A 4-step approach was used to test the mediation effect of internet addiction [71,72]. First, the direct effect of vertical individualism was significant (β=.27, SE=.05, CR=5.79, *P*<.001), whereas the direct effect of horizontal individualism was not (β=–.40, SE=.06, CR=–1.04, *P*=.30). Second, the direct effects of vertical individualism (β=.20, SE=.02, CR=4.57, *P*<.001) and horizontal individualism (β=–.13, SE=.03, CR=–3.16, *P*<.001) on cyberbullying were significant. Third, the direct effect of internet addiction on cyberbullying was significant (β=.41, SE=.02, CR=9.92, *P*<.001). Finally, the Sobel test showed that the indirect effect of vertical individualism on cyberbullying via the mediator (ie, internet addiction) was significant (CR=5.87, SE=.06, *P*<.001). These results support partial mediation and indicate that vertical individualism has a significant effect on cyberbullying through internet addiction.

### Gender Differences

One-way multivariate analysis of variance was used to investigate gender differences between women (462/665) and men (203/665) on vertical versus horizontal individualism. Results showed a significant difference between women and men, where men (mean 26.45 points, SD 6.12 points) scored significantly higher than women (mean 25.04 points, SD 5.66 points) in vertical individualism (F_2,662_=6.42, *P*=.002, Wilk’s λ=.98, partial *η*^2^=.02). However, the effect size of gender on vertical individualism, while statistically significant, was practically minimal (partial eta-squared=.02). Multivariate analysis of variance results also showed a significant difference in internet addiction between women and men (F_5,659_=7.97, *P*<.001, Wilk’s λ=.94, partial *η*^2^=.06), where men scored higher than women. Further, independent samples *t*-test results suggested no gender difference in cyberbullying between men and women (t_663_=–1.32, *P*=.19). It is important that a previous study [30] suggested significant differences in internet addiction among age groups. However, the sample in this current study was comprised of a restricted age group (17-19 years old); therefore, we were not able to investigate the differences among age groups.

## Discussion

In this research, we investigated the relationships among vertical versus horizontal individualism, cyberbullying, and internet addiction via a theoretical model and provided valuable implications for mental health professionals and researchers. Arpaci et al [73] suggested that individualists face higher socialization problems and the risk of failure in communicating with others. Consequently, they prefer alternative environments, such as cyberspace. Therefore, they are expected to be more prone to internet addiction. Accordingly, we expected significant relationships among individualism, cyberbullying, and internet addiction. Our results showed a stronger correlation between internet use and vertical individualism than between internet use and horizontal individualism. This suggests that vertical individualists tend to spend more hours on the internet and thereby, are more vulnerable to internet addiction.

We found a significant intervening effect of internet addiction in the association between vertical individualism and cyberbullying. These results support the important intervening role of internet addiction in the relationship between vertical individualism and cyberbullying. Thus, knowing the individual’s cultural orientation and level of internet addiction might be helpful in the prevention or treatment of their cyberbullying behaviors.

Self-reliance and uniqueness are some of the relatively more positive characteristics of horizontal individualists [74], while competition and hedonism are more of the destructive characteristics of vertical individualists [45]. Thus, we expected that vertical individualism would positively predict cyberbullying. Our findings support that vertical and horizontal individualism are significant positive and negative predictors of cyberbullying, respectively. In general, those who score higher for individualism foster a propensity to avoid establishing close relationships with others and gradually withdraw from society and social environments [75]. In addition, these individuals do not take the initiative to solve problems but are more likely to evade responsibility. As a result, research has associated individualism with problematic behaviors [35,46]. However, we investigated the specific effects of vertical versus horizontal individualism, which have not been studied previously. Based on our findings, we conclude that those individuals who seek distinctiveness and are especially concerned with comparing themselves to others (ie, vertical individualists) tend to show more cyberbullying tendencies. On the contrary, individuals who are more self-reliant and seek individuality (ie, horizontal individualists) show lower cyberbullying tendencies. However, the reader should also keep in mind that both predictions have relatively small effect sizes, meaning that although the predictions are statistically significant, their effects are minimal. In short, we conclude that vertical individualists tend to develop cyberbullying behaviors depending on their level of internet addiction. The points raised in this study should be considered when prevention or treatment programs are being developed for cyberbullying. Different treatment approaches to cyberbullying should be employed depending upon whether the individual is vertically or horizontally oriented and his or her level of internet addiction.

These results also indicate that vertical, but not horizontal, individualism is a significant predictor of internet addiction. Previous research indicates an association between individualism and addictive behaviors [33,34]; however, our results provide details of such a relationship. We conclude that individualists who prefer gratification through competition and personal achievement are under greater risk of internet addiction, not those who tend to just value uniqueness with equality.

The findings of our research confirm the findings of Casas et al [18] who concluded that cyberbullying is affected by internet addiction. We found that higher internet addiction levels predict higher cyberbullying behaviors. Therefore, it seems reasonable to assume that addicts will show higher levels of cyberbullying. This conclusion is consistent with previous literature [29,50,76-78].

The findings show that vertical individualism has a significant effect on internet addiction. More interestingly, the findings suggest that vertical individualists are more vulnerable to internet addiction. Further, the findings indicate a significant relationship between internet addiction and cyberbullying. Therefore, prevention programs for cyberbullying should take cultural orientations into account.

Although vertical and horizontal individualism were perceived to be dichotomous rather than orthogonal, the correlation analysis results shown in Table 2 suggest a positive correlation between both types of individualism. It is important that both horizontal and vertical individualists focus on an autonomous self-concept; however, horizontal individualists place a strong emphasis on equality in status, whereas vertical individualists accept inequalities [8].

Although this study is highly original, it has several limitations. First, the sample used in the study was comprised of a restricted age group and thus homogenous; therefore, the theoretical model needs further confirmation across different age groups. Crosscultural studies should be conducted in different cultures to improve the external validity of the findings. Second, cultural orientations are not the only predictors of cyberbullying, nor is internet addiction the only mediator. However, based on the literature, this study used a single exogenous factor, vertical versus horizontal individualism, and a single mediator, internet addiction, in the structural model. Many other dispositional or situational factors and mediators would be equally worthwhile to explore in future studies. Further, there may be several equivalent models that can predict the impact of both cultural orientations or internet addiction on cyberbullying. This suggests that the proposed model is certainly supported, but not proven; therefore, further studies should replicate the research model.

In this study, vertical and horizontal individualism were studied as cultural orientations of the participants, and they were measured using an individual-level measurement within a monocultural sample. In the same vein, others studied and operationalized the same orientations at the individual level. For example, Bourgeois [79] argued that vertical and horizontal collectivism-individualism are testable dimensions of culture at the individual level. He investigated values (ie freedom and equality) of vertical-horizontal individualists and collectivists by collecting data from active party members of the Republican and Democratic parties in New Brunswick, Canada. Similarly, Le [80] investigated the relationships among vertical individualism, narcissism, immature love, and ludus based on data collected from 179 undergraduate students at University of California Davis. The results suggested that vertical individualism has a positive significant effect on narcissism and immature love. The findings suggested that vertical individualists were more prone to ludic love style and saw others as fulfillment of wishes and needs.

Finally, previous literature perceived vertical and horizontal individualism to be orthogonal [11]. However, our results indicate a positive correlation between vertical and horizontal individualism, suggesting a more complex relationship. Confirming this, Triandis [11] argued that, along with the horizontal-vertical dimension, there are many other dimensions defining different varieties of collectivism and individualism.

## Abbreviations

AGFI: adjusted goodness of fit index.
CFI: comparative fit index.
GFI: goodness of fit index.
IFI: incremental fit index.
NFI: normed fit index.
RMR: root mean square residual.
RMSEA: root mean square error of approximation.
SRMR: standardized root mean square residual.
TLI: Tucker Lewis index.
